# Predictors for readmission due to cellulitis among Japanese patients

**DOI:** 10.1111/1346-8138.15771

**Published:** 2021-02-01

**Authors:** Yuta Norimatsu, Yuki Ohno

**Affiliations:** ^1^ Department of Dermatology JR Tokyo General Hospital Tokyo Japan; ^2^ Department of Dermatology University of Tokyo Graduate School of Medicine Tokyo Japan

**Keywords:** cellulitis, hyperlipidemia, hypertension, hypoalbuminemia, lymphedema

## Abstract

Cellulitis is an infection of the soft tissues of the skin. Some patients are hospitalized multiple times; lymphedema, chronic venous insufficiency, peripheral circulatory disturbance, and deep vein thrombosis are said to be risk factors for multiple admissions. Conversely, in Japan, elderly women and undernourished elderly have been reported to be at risk of multiple hospitalizations, suggesting a different patient background from that reported overseas. We investigated the characteristics of readmission cases for patients hospitalized for cellulitis in Japan. We retrospectively examined cases of cellulitis and erysipelas admitted between April 1, 2005 and March 31, 2018. Patients with cellulitis or erysipelas at multiple sites and those with osteomyelitis, pressure ulcer infection, and necrotizing fasciitis were excluded. In terms of recurrence, only hospitalizations for recurrence at the same site were considered. Patients with multiple hospitalizations had significantly lower blood albumin levels than those hospitalized only once. Furthermore, the rates of lymphedema, hypertension, and hyperlipidemia were significantly higher in patients hospitalized multiple times. Other laboratory and clinical factors were not statistically significant. Therefore, hypoalbuminemia with or without liver dysfunction, lymphedema, hypertension, and hyperlipidemia were suggested as risk factors for cellulitis recurrence. However, chronic venous insufficiency, peripheral circulatory disturbance, and deep vein thrombosis did not seem to be risk factors in Japanese cases. Japanese cases had a low body mass index (approximately 25 kg/m^2^), suggesting that the patient background may be different from that in existing reports. This suggests that the risk factors in Japanese cases may be different from those reported in other countries.

## 1 INTRODUCTION

Cellulitis is a skin and soft tissue infection that is accompanied by symptoms, such as swelling, flushing, and pain [1]. The main causative bacterial species are group A β‐*Streptococcus* and *Staphylococcus aureus* [2]. Some patients, especially those with chronic swelling (lymphedema), obesity, and diabetes, are at an increased risk of cellulitis [1, 3]. Previous studies have reported that 41% to 45.3% of cellulitis cases eventually recur [4].

A recurrence prediction score for the cellulitis of the lower limbs has been developed, and patients with lymphedema, chronic venous insufficiency, peripheral circulatory disturbance, and deep vein thrombosis are considered to be at risk [4]. While this score is expected to be applied clinically, it has limited usefulness because it is limited to the lower limbs, which are not necessarily examined at the time of admission.

According to a report from Japan, among patients with cellulitis, recurrence is more common in elderly women, and malnutrition was indicated as a possible reason [5]. Serum albumin, a serum protein, is known as an index for nutritional disorders independent of age [6]. Furthermore, hypoalbuminemia is a known risk factor for cellulitis in patients with cirrhosis [7]. Blood albumin concentration is known to be associated with liver function [8]; therefore, deterioration of liver function may affect albumin levels. However, patients with recurrence of cellulitis are not the only patients with cirrhosis. We investigated all hospitalized cases of cellulitis, including all cases requiring hospitalization due to cellulitis recurrence.

In this study, we examined whether differences in blood tests and clinical findings at the time of first admission could be observed between single and multiple admission cases of patients hospitalized due to cellulitis at a Japanese hospital.

## 2 METHODS

We retrospectively examined cases of cellulitis and erysipelas hospitalization between April 1, 2005 and March 31, 2018. Cases with cellulitis or erysipelas at different sites were excluded. In cases with recurrence, only hospitalizations for recurrence at the same site were considered. Patients with osteomyelitis, pressure ulcer infection, and necrotizing fasciitis were excluded.

For statistical analysis, we used the Mann‐Whitney *U* test, Fisher's exact test, and Pearson's correlation coefficient. A *P*‐value of <0.05 was considered statistically significant. Prism 8 (GraphPad Software, San Diego, CA, USA) was used for statistical analysis.

## 3 RESULTS

Tables 1, 2, 3 compare the characteristics of the single admission group with those of the multiple admissions group at their first admission. Table 1 shows the patient characteristics of the two groups. The rates of lymphedema, hypertension, and hyperlipidemia were significantly higher in patients hospitalized multiple times than in patients hospitalized only once. Table 2 shows the single and multiple admissions groups’ vital signs and clinical findings, which were not statistically significant. Table 3 compares the blood test results of the single and multiple admissions groups. Patients with multiple hospitalizations had significantly lower blood albumin levels than those who were hospitalized only once.

**Table 1:** Characteristics of patients. Fisher's exact test or the Mann‐Whitney U test was used for statistics.

Patient characteristics	Multiple admissions (n = 16) (mean ±SD)	Single admission (n = 181) (mean ±SD)	*P*‐Value
Age	59.38 ± 18.88	52.68 ± 18.69	0.1867
Sex (Male: Female)	10:6	125:56	0.5837
Body Mass Index (kg/m^2^)	25.93 ± 7.531	25.92 ± 6.535	0.8808
Weight (kg)	71.59 ± 26.07	71.38 ± 20.66	0.9001
*Medical history*			
Liver dysfunction (+:−)	1:15	7:171	0.5044
Diabetes mellitus (+:−)	6:10	45:135	0.3706
Hypertension (+:−)	9:7	49:131	0.0216
Hyperlipidemia (+:−)	6:10	22:158	0.0144
Thrombosis (+:−)	0:16	3:177	>0.9999
Cancer (+:−)	4:12	16:164	0.0643
Autoimmune disease (+:−)	0:16	5:175	>0.9999
Peripheral vascular disease (+:−)	0:16	1:77	>0.9999
Chronic venous insufficiency (+:−)	0:16	3:75	0.4890
Lymphedema (+:−)	7:9	10:170	<0.0001
Immunosuppressant use (+:−)	0:16	8:172	>0.9999

SD, standard deviation.

**Table 2:** Vital signs and clinical findings of patients. Fisher's exact test or the Mann‐Whitney U test was used for statistics.

Vital signs and clinical findings	Multiple admissions (n = 16) (mean ± SD)	Single admission (n = 181) (mean ± SD)	*P*‐Value
Body temperature (℃)	37.23 ± 0.9084	36.99 ± 0.9205	0.3439
Heart rate (bpm)	82.8 ± 12.17	84.06 ± 16.56	0.8963
Oxygen saturation (%)	97.0 ± 2.646	97.62 ± 1.577	0.9540
Respiratory rate (/minute)	20.5 ± 5.447	22.5 ± 9.000	>0.9999
Systolic arterial pressure (mmHg)	125.6 ± 13.07	134.9 ± 77.63	0.4821
Pain (+:−)	9:5	143:26	0.0652
Acute renal dysfunction (+:−)	1:14	6:153	0.4742
Skin damage (+:−)	7:9	112:67	0.1815
Blisters, purpura, snowgrasping sense (+:−)	1:15	21:153	0.6991

SD, standard deviation.

**Table 3:** Blood test results of patients. The Mann‐Whitney U test was used for statistics.

Blood test (normal range)	Multiple admissions (n = 16) (mean ± SD)	Single admission (n = 181) (mean ± SD)	*P*‐Value
*Hemogram*			
White blood cells (/μL) (3500–9200)	10 263 ± 3941	10 505 ± 4316	0.9125
Red blood cells (×10^6^/μL) (3.84–5.54)	4.253 ± 0.7505	4.392 ± 0.6309	0.4170
Platelets (/μL) (155 000–365 000)	236 400 ± 86 800	250 400 ± 83 570	0.5917
Hemoglobin (g/dL) (11.3–16.6)	13.24 ± 2.144	13.52 ± 1.734	0.6923
*Electrolyte*			
Sodium (mEq/L) (136–146)	137.6 ± 3.931	138.3 ± 3.328	0.5753
Potassium (mEq/L) (3.5–5.4)	4.145 ± 0.4741	4.037 ± 0.3882	0.6487
Chloride (mEq/L) (96–108)	102.4 ± 3.931	102.3 ± 3.642	0.9671
Renal function			
Blood urea nitrogen (mg/dL) (8–22)	23.68 ± 29.93	14.42 ± 5.799	0.1910
Creatinine (mg/dL) (0.35–1.11)	1.229 ± 1.581	0.8441 ± 0.2592	0.6248
*Liver function*			
Total bilirubin (mg/dL) (0.3–1.2)	0.7385 ± 0.4407	0.7807 ± 0.4863	0.7762
Aspartate transaminase (U/L) (8–38)	32.00 ± 21.74	28.98 ± 18.84	0.2943
Alanine transaminase (U/L) (4–44)	29.81 ± 23.19	29.32 ± 22.66	0.6589
γ‐glutamyl transpeptidase (U/L) (16–84)	42.42 ± 27.83	53.67 ± 58.64	0.8930
*Diabetes and coagulation*			
Glucose (mg/dL) (65–110)	170.7 ± 78.73	148.7 ± 91.18	0.3618
Hemoglobin A1c (%) (4.3–5.8)	6.383 ± 1.026	6.824 ± 1.915	0.8688
Fibrinogen (mg/dL) (~400)	–	583.2 ± 174.5	–
*Nutrition and inflammation and others*			
Total protein (g/dL) (6.3–8.1)	7.343 ± 0.7635	7.086 ± 0.6719	0.4771
Albumin (g/dL) (3.9–5.1)	3.592 ± 0.4814	3.869 ± 0.5378	0.0428
Lactate Dehydrogenase (U/L) (119–229)	250.8 ± 58.03	237.6 ± 69.80	0.3022
Creatine kinase (U/L) (61–255)	161.3 ± 179.3	151.6 ± 243.6	0.7926
Procalcitonin (ng/mL) (~0.5)	–	0.09 ± 0.05657	–
Antistreptolysin‐O (IU/mL) (~200)	385.4 ± 456.0	188.1 ± 200.6	0.3261
C‐reactive protein (mg/dL) (~0.3)	8.368 ± 7.191	7.693 ± 7.307	0.6364

SD, standard deviation.

## 4 DISCUSSION

We examined the possible differences in patient characteristics, vital signs, clinical findings, and blood tests between the single and multiple admission groups. Serum albumin levels were significantly lower in the multiple admissions group. Furthermore, the rates of lymphedema, hypertension, and hyperlipidemia were significantly higher in the multiple admission group than in the single admission group.

Lymphedema has been suggested as a risk factor for multiple hospitalizations for cellulitis in previous reports [3, 4, 9]. In contrast, our study showed no significant difference between the two groups in terms of chronic venous insufficiency, peripheral circulatory disturbance, and deep vein thrombosis. Lymphedema has been suggested to be significantly more prevalent in patients aged 75 years or more, but chronic venous insufficiency, peripheral circulatory disturbance, and deep vein thrombosis have not been suggested to differ significantly with age. Kumar et al. reported that patients with cellulitis under and over the age of 75 years had body mass indexes (BMIs) of 36.0 ± 12.3 kg/m^2^ and 28.3 ± 8.0 kg/m^2^, respectively, with a significant difference at *P* < 0.001 [10]. In this study, there was no significant difference of approximately 25 in both single and multiple hospitalization cases. This suggests that obesity is not as common among Japanese patients with cellulitis as in patients with cellulitis in other countries.

Hypertension is said to be significantly higher in cellulitis patients aged 75 years or more. As mentioned earlier, the BMI of patients over 75 years is significantly lower than that of patients below 75 years. This suggests that hypertension is an important risk factor in patients with low BMI. Hypertension is considered an important risk factor because BMI was not high in Japanese cases. In contrast, hypertension does not pose a risk of multiple hospitalizations in patients with lymphedema [9]. Hypertension is said to be a risk factor for lymphedema [11]. Therefore, it is possible that there was no significant difference in studies involving only lymphedema. In Japan, systolic blood pressure has been declining in all age groups in recent decades, and there are less than 6% of hypertensive patients in any age group [12]. Accordingly, it is considered that hypertension is a factor independent of age in Japan.

Although an association between hyperlipidemia and skin infections has been suggested in previous studies, it is unclear whether it is a risk factor for recurrence [13]. This study suggests that hyperlipidemia is also a risk factor for the recurrence of cellulitis in Japanese patients.

In previous reports, hypoalbuminemia was reported as a risk factor for cellulitis recurrence in patients with cirrhosis [7]. However, in our study, there was no difference between the multiple and single admission groups in terms of the presence or absence of a medical history of liver dysfunction.

Aspartate transaminase, alanine transaminase, and γ‐glutamyl transpeptidase levels were also not different between the multiple and single admission groups. This suggests that the presence or absence of liver dysfunction on admission was not associated with recurrence, but it may also suggest that malnutrition is involved in recurrence. However, in this study, the mechanism is unknown; therefore, further study is required in the future.

A limitation of this study is that it is a single‐center retrospective study. Additionally, JR Tokyo General Hospital is a hospital that plays a central role in the region, and patients who have visited the hospital even once are examined at this hospital. However, in Japan, patients are free to choose the hospital to visit; therefore, it is undeniable that they may not have reported that they have been admitted to another hospital for some reason.

In conclusion, hypoalbuminemia was suggested as a risk factor for recurrence of cellulitis with or without liver dysfunction. Lymphedema, hypertension, and hyperlipidemia were also shown to be possible risk factors for cellulitis recurrence, but this study suggests that chronic venous insufficiency, peripheral circulatory disturbance, and deep vein thrombosis were not risk factors in Japanese cases. Compared with existing reports, Japanese patients had a low BMI of about 25 kg/m^2^, suggesting a difference in patient background. This suggests that the risk factors in Japanese cases may be different from those reported in other countries.

## CONFLICT OF INTEREST

The authors declare no conflict of interest.

## ETHICAL CONSIDERATIONS

All study participants provided informed consent, and the study design was approved by the JR Tokyo General Hospital's ethics review board. This study complies with the Declaration of Helsinki.
